# Thermometry of bosonic mixtures in Optical Lattices via Demixing

**DOI:** 10.1038/s41598-017-05353-6

**Published:** 2017-07-11

**Authors:** F. Lingua, B. Capogrosso-Sansone, F. Minardi, V. Penna

**Affiliations:** 1 Department of Applied Science and Technology and u.d.r. CNISM, Politecnico di Torino, I-10129 Torino, Italy; 2 Department of Physics, Clark University, Worcester, Massachusetts 01610 USA; 3 Istituto Nazionale di Ottica (INO-CNR), I-50019 Sesto Fiorentino, Italy; 4 European Laboratory for Non-Linear Spectroscopy (LENS) and Dipartimento di Fisica, Università di Firenze, I-50019 Sesto Fiorentino, Italy

## Abstract

Motivated by recent experiments and theoretical investigations on binary mixtures, we investigate the miscible-immiscible transition at finite temperature by means of Quantum Monte Carlo. Based on the observation that the segregated phase is strongly affected by temperature, we propose to use the degree of demixing for thermometry of a binary bosonic mixture trapped in an optical lattice. We show that the proposed method is especially sensitive at low temperatures, of the order of the tunnelling amplitude, and therefore is particularly suitable in the regime where quantum magnetism is expected.

## Introduction

Ultracold atoms in optical lattices are generally regarded as an almost ideal experimental setting to investigate many-body quantum physics in strongly correlated regimes^1^. However, next to undisputed strengths, atomic systems suffer from some notable limitations. Perhaps surprisingly if compared to condensed-matter counterparts, for strongly correlated quantum gases measuring fundamental parameters, such as temperature, is far from trivial, a fact often encumbering the comparison between theoretical and experimental findings. The physical reason is that the primary thermometric quantity used in cold atoms experiments, namely the momentum distribution, in strongly correlated regimes is often dominated by quantum rather than thermal fluctuations, thereby becoming quite insensitive to temperature variations. In recognition of its importance, thermometry for optical lattices have sparked numerous theoretical proposals and experiments^2^. Quite generally, two main approaches have been pursued for thermometry: through ancillary samples (or sample subsets) in a well-understood, e.g. weakly interacting (WI), regime^3–7^; or by measuring *in*-*situ* local density fluctuations with high-resolution imaging^8–13^. Other proposed methods still await experimental demonstration^14–16^.

### A new thermometric scheme

In this work, we propose a thermometry technique for ultracold quantum mixtures in optical lattices based on the demixing of two mutually repulsive components. Multi-components BECs have been created long ago^17, 18^, while recently seminal works in thermometry have been reported both with bosonic and fermionic samples. With a rubidium condensate, demixing between two spin components was induced by a magnetic field gradient and the width of the interface region was used to estimate the temperature^3, 4^. Recently, the spin waves, or ‘magnons’, in a spinor Rb condensate were used to reduce the entropy per particle to values as low as 0.02 *k*_*B*_^19^, an order of magnitude below the values required for the onset of magnetic phases^20^. On the fermionic side, anti-ferromagnetic correlations have been detected by means of Bragg scattering^21^, and also, very recently, directly by means of single-site imaging^22–24^.

We analyze the effect of temperature fluctuations on a demixed phase of a mixture of two bosonic species trapped in an optical lattice with and without an external harmonic confinement. Demixing, i.e. the spatial separation of the two components, occurs when inter-species repulsion overcomes the intra-species one^25–28^. An important role is played by temperature fluctuations which compete with, and eventually destroy, demixing^29^. Here, we take advantage of this competition to propose a route for thermometry in ultracold mixtures: we employ a suitable global estimator of demixing which can readily be measured and used to determine the temperature.

We first consider the case of a moderately shallow optical lattice, where each species is superfluid. At sufficiently weak interspecies interaction, this regime is interesting as the momentum distribution of either species allows an independent determination of the temperature^30^ to validate the currently proposed thermometry. In the case of a deeper optical lattice, thus a strongly-interacting (SI) regime^31^, we lack reliable temperature estimators other than the direct microscopic observation of particle-hole pairs. In this regime, our proposed thermometry proves a remarkably effective tool. Specifically, we show how the degree of demixing characterizing the spatial distribution of trapped mixtures depends upon the temperature. In particular, demixing in low density regions is more susceptible to temperature fluctuations than in high density regions.

In this paper, we also probe the effect of temperature on spatial distribution, showing that temperature-induced changes in the shell-structure of the trapped density correspond to a dramatic signature in the boson interference patterns produced when the confining potential is turned off. Our results are based on large-scale path-integral quantum Monte Carlo simulations by a two-worm algorithm^32^.

### The Model and the phase diagram

**Figure 1:**
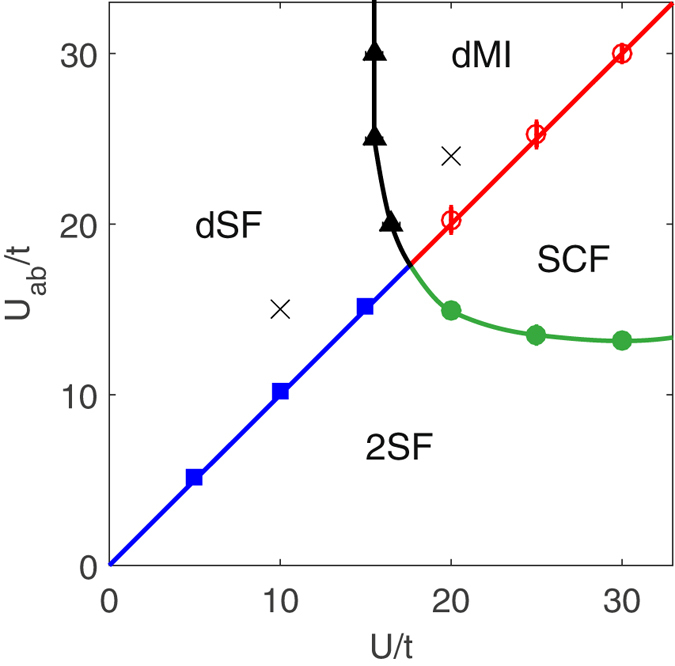
Ground-state phase diagram of twin bosonic species at total filling *n* = 1, from ref. 29. Crosses indicate the parameters used here for simulations. The dSF (dMI) phase represents two spatially separated superfluids (Mott-Insulators). 2SF describes two mixed superfluids, and SCF is a global Mott-insulator phase where mobility is allowed as a superflow in the particle-hole channel.

A mixture of two *bosonic* species trapped in a two-dimensional square optical lattice, is described by the two-component Bose-Hubbard (BH) model:

1 $$H={H}_{a}+{H}_{b}+{U}_{ab}\sum _{i}\,{n}_{ai}{n}_{bi}$$

where *U*_*ab*_ is the inter-species repulsion, *n*_*ai*_, *n*_*bi*_ are the number operators at site *i* for species A and B respectively, and

2 $${H}_{c}=\frac{{U}_{c}}{2}\sum _{i}\,{n}_{ci}({n}_{ci}-\mathrm{1)}-{t}_{c}\sum _{\langle ij\rangle }\,{c}_{i}^{\dagger }{c}_{j}-\sum _{i}\,{\mu }_{ci}{n}_{ci},$$

where 〈*ij*〉 denotes to sum over nearest neighboring sites, *c* = *a*, *b* the bosonic species, *c*_*i*_ (${c}_{i}^{\dagger }$) the annihilation (creation) operators satisfying $[{c}_{i},{c}_{i}^{\dagger }]=1$, *U*_*c*_ the intra-species repulsion, *t*_*c*_ the hopping amplitude, and *μ*_*ci*_ = *μ*_*c*_ (${\mu }_{ci}={\mu }_{c}-{\omega }_{H}{{\overrightarrow{r}}_{i}}^{2}$) the chemical potentials in the homogeneous (trapped) case. In this paper, for simplicity, we consider a model with twin species, namely, we set *U*_*a*_ = *U*_*b*_ = *U*, *t*_*a*_ = *t*_*b*_ = *t* and *μ*_*a*_ = *μ*_*b*_ = *μ*. The condition on the chemical potentials implies that *N*_*a*_ = *N*_*b*_, where *N*_*a*_ and *N*_*b*_ are the total number of particles of species *A* and *B*, respectively. The ground-state phase diagram of a twin-species mixture at total integer filling features a demixed superfluid (dSF), or a demixed Mott-insulator (dMI), when the interspecies interaction becomes greater than the intraspecies repulsion, and a double-superfluid phase (2SF) or a supercounterflow (SCF) otherwise. This is illustrated in Fig. 1 where integer total filling factor *n* = 1 has been assumed.

## Results

We first consider a homogeneous system of linear size *L* (in unit of the lattice step *d* which we set as our unit length) with periodic boundary conditions. We work at integer total filling factor considering both *n* = 1 and *n* = 2. This corresponds to the condition ${N}_{a}+{N}_{b}=n{L}^{2}$. Then we move to analyse a trapped system by introducing a harmonic trapping potential.

To study the effect of temperature fluctuations, we first focus on the transition 2SF-dSF since this can be observed for any choice of the filling factor^29^, while the transition from dMI-SCF requires an integer value of the filling factor.

We quantify demixing effects through the parameter

3 $${\rm{\Delta }}=\frac{1}{M}\sum _{i}\,{[\frac{\langle {n}_{ai}\rangle -\langle {n}_{bi}\rangle }{\langle {n}_{ai}\rangle +\langle {n}_{bi}\rangle }]}^{2},$$

where the sum runs over the *M* = *L* ^2^ lattice sites; evidently, Δ ranges from 0, if all sites are equally populated, to 1, for complete demixing.

### Homogeneous case

**Figure 2:**
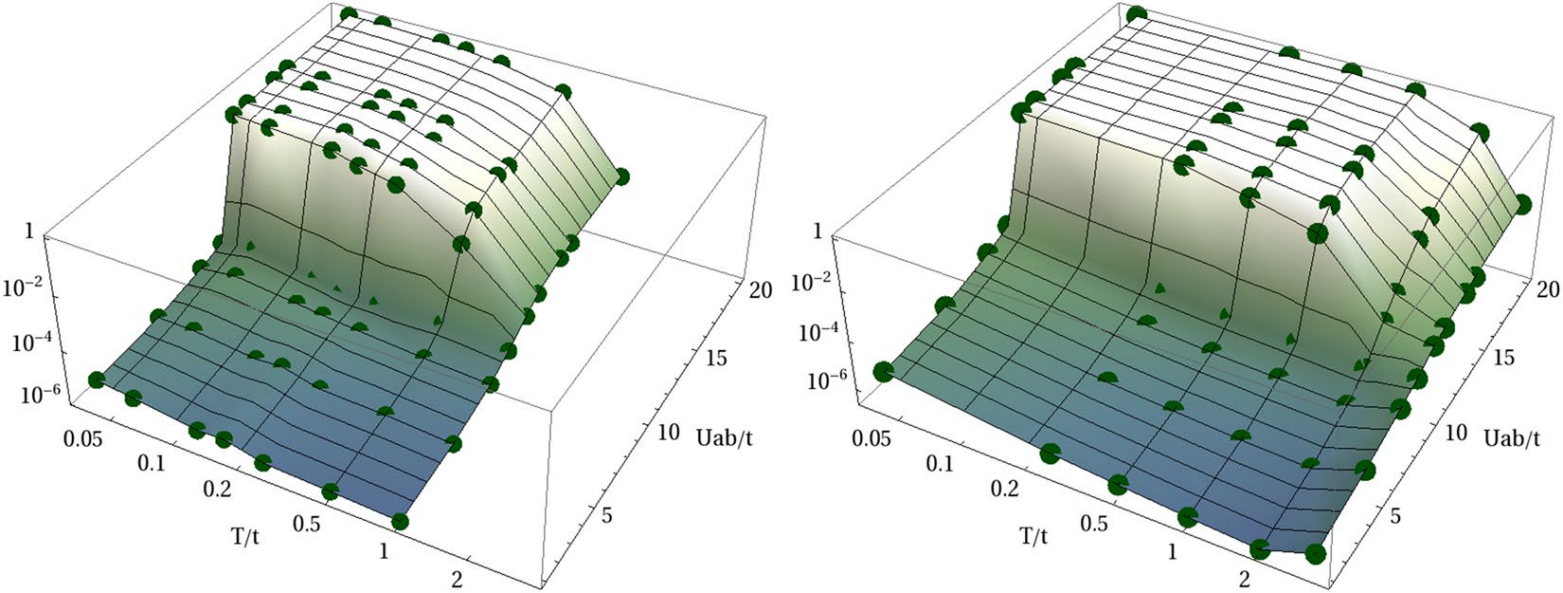
Parameter Δ as a function of temperature *T*/*t* and interspecies interactions *U*_*ab*_/*t*, at *U*/*t* = 10. Total filling *n* = 1 (left panel) and *n* = 2 (right panel). Points show numerical results.

Figure 2 shows Δ as a function of temperature *T* and inter-species interactions *U*_*ab*_, at fixed *U* = 10*t*, for total filling *n* = 1 (upper panel) and *n* = 2 (lower panel). At lower temperatures, a step-like increase in the value of the demixing parameter Δ signals the onset of strong demixing in the system. As the temperature is increased, thermal fluctuations become more prominent and the mixing of the two components is restored even for *U*_*ab*_ > *U*. We observe a pronounced dependence of Δ on temperature in the dSF phase, with a three order of magnitudes drop within a range of temperatures of the order of the tunneling energy *t*. On the contrary, in the 2SF phase, Δ is rather insensitive to the temperature, and it remains orders of magnitude smaller than in the dSF phase. As outlined below, the strong dependence of Δ on *T* displayed in the dSF phase motivates the basic idea of extracting the temperature from the measurement of the demixing parameter.

For total filling factor *n* = 2 (lower panel in Fig. 2), the larger energy penalty associated with the overlap between components leads to a more evident demixing^29^. Robustness against miscibility results in a stronger robustness against temperature fluctuations than at lower filling factors: notice the evident increase in temperatures needed in order to destroy demixing for *n* = 2. In this sense, larger filling factors shift the operating range of the proposed thermometer towards higher temperatures. This behavior can be understood by observing that, for *U*_*ab*_ > *U*, increasing the filling factor further inhibits the start of the mixing process of the two components. In this sense, the minimal mixing consists in displacing a single boson *a* (*b*) in the domain of components B (A). For *n* = 2, the heuristic calculation of the free-energy cost for creating a double pair *ab* gives ${\rm{\Delta }}F=\mathrm{2(}{U}_{ab}-U)(n-1)-T{k}_{B}\,\mathrm{ln}({L}^{4}\mathrm{/4)}$. The mixing temperature is found to be ${k}_{B}T=\mathrm{2(}{U}_{ab}-U)/\mathrm{ln}({L}^{4}\mathrm{/4)}$. For *n* = 1, the mixing process begins with the formation of a single pair *ab* and a hole. This entails ${\rm{\Delta }}F={U}_{ab}-T{k}_{B}\,\mathrm{ln}({L}^{4}\mathrm{/4)}$ and ${k}_{B}T={U}_{ab}/\mathrm{ln}({L}^{4}\mathrm{/4)}$. In both cases, using the parameter values of Fig. 2 gives *k*_*B*_ *T*/*t* ~ 1 in agreement with numerical results. The dependence on the lattice size *L* reflects the finite-size character of our model. The temperature at which the first pairs *ab* crop up is proportional to *U*_*ab*_, thus confirming the inhibition of the mixing effect for increasing *U*_*ab*_.

### Trapped system

In order to consider a more general and realistic scenario, we relax the homogeneity assumption and study the system in a harmonic trap. The chemical potential of species *c* = *a*, *b*, transforms according to

4 $${\mu }_{ci}={\mu }_{c}-{\omega }_{H}^{2}{\overrightarrow{r}}_{i}^{\,2}$$

where *ω*_*H*_ is the curvature of the harmonic trap and ${\overrightarrow{r}}_{i}$ the position vector of lattice site *i*. This leads to a site-dependent filling factor *n*_*i*_. Generally, demixing is not affected by the presence of a harmonic potential as far as the condition *U*_*ab*_ > *U* is satisfied. Demixing in the trap manifests itself through the occurrence of a sharp and straight boundary between the two species. This represents the minimum-energy configuration for a demixed system in a trap, as originally predicted for continuous systems^25^.

#### Weak Interaction

**Figure 3:**
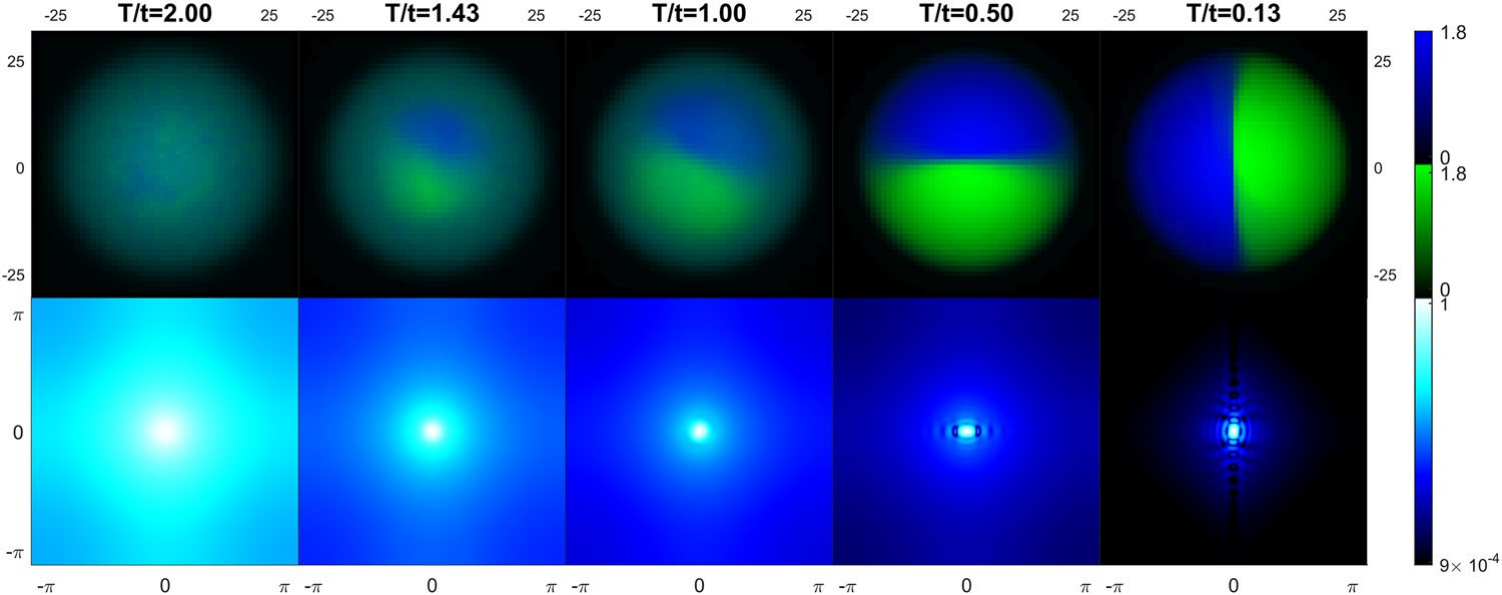
Density maps (first row) of species A (green) and B (blue), and computed momentum distributions of species B (second row) for decreasing temperature (left to right). The mixture (*U*/*t* = 10, *U*_*ab*_/*t* = 15) is trapped in a harmonic potential of strength *ω*_*H*_/*t* = 0.03.

In the following, we present finite temperature results for the trapped case of WI mixtures. We find that a spatial shell structure arises at intermediate temperatures (see Fig. 3), in which a central demixed phase (dSF) is surrounded by a shell of mixed phase (2SF). In the first row of Fig. 3, it is well visible that the temperature-induced mixing effect first appears where the density is lower (that is in the outer shell) and in the proximity of the boundary separating the two species. Such an effect is due to the larger entropy associated with demixing in these regions. For sufficiently large temperatures we detected the presence of a third surrounding shell of a Normal-Fluid (NF) phase. As expected, the thickness of the NF shell increases for increasing temperatures (see Supplementary information for details).

**Figure 4:**
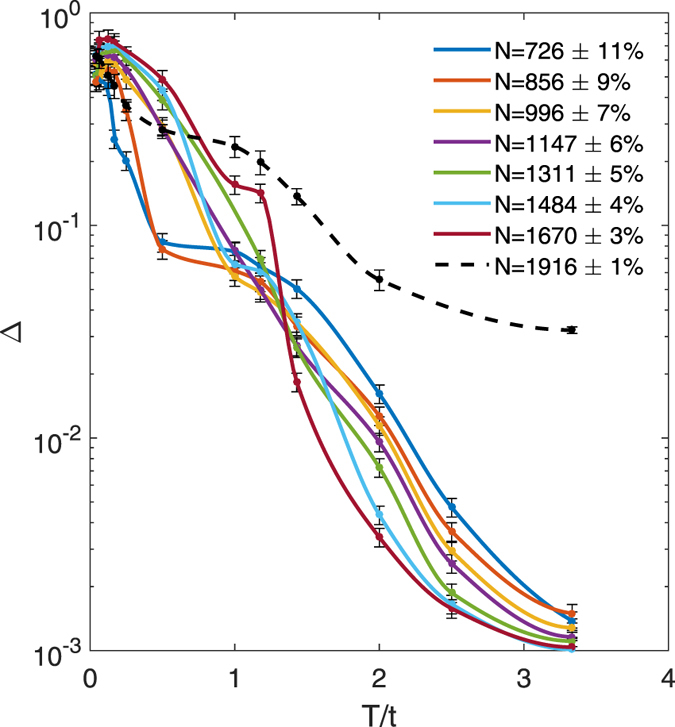
Δ parameter as a function of temperature in the trapped case. Solid lines: ${\omega }_{H}/t=0.03$, $U/t=10$, ${U}_{ab}/t=15$; black dashed line: and ${\omega }_{H}/t=0.12$, $U/t=20$, ${U}_{ab}/t=24$.

In Fig. 4 we show the behaviour of parameter Δ as a function of *T*/*t* for the trapped case. We have performed simulations for different number of atoms *N*, both in the WI (solid lines) and SI (black dashed line) regimes. In the WI regime with *U*/*t* = 10 and *U*_*ab*_/*t* = 15, besides confirming that a system with a higher number of bosons is more robust against temperature mixing, we notice that, over the considered range of temperature, the demixing parameter drops approximately one order of magnitude less than in the homogeneous case. Larger local densities at the center of the trap imply larger interactions energies thus reducing the boson mobility and their mixing degree.

In search of additional experimental signatures of the temperature-driven transition from the dSF to the 2SF phase, we computed the momentum distributions ${n}_{c,{\bf{k}}}={|{\varphi }_{({\bf{k}})}|}^{2}{\sum }_{i,j}\,{e}^{i{\bf{k}}({{\bf{r}}}_{i}-{{\bf{r}}}_{j})}\langle {c}_{i}^{\dagger }{c}_{j}\rangle$ ^33^ for species *c* = *a*, *b*. Distributions *n*_*c*,**k**_, integrated along one direction, are recorded by the time-of-flight images experimentally observed, provided that interactions have a negligible effect during the expansion. In the second row of Fig. 3 we plot the **k**-periodic part of momentum distribution, i.e. ${\tilde{n}}_{c}({\bf{k}})\equiv {n}_{c}({\bf{k}})/{|{\varphi }_{({\bf{k}})}|}^{2}$, for species B (due to the symmetry of the system the momentum distributions of species A show the same features). For increasing temperatures (right to left), we observe how the appearance of the spatial shell structure is accompanied by changes in the momentum distribution. When the “hard-wall” separating the demixed species is present, the phase coherence of each species is restricted to the portions of the lattice where the species is confined, and this produces the fringes shown in the ${\tilde{n}}_{c}({\bf{k}})$ images of Fig. 3. Such fringes arise due to the interference of waves bouncing back from the “hard-wall” separating the demixed species.

#### Strong Interaction

After showing that parameter Δ is a convenient temperature indicator for WI mixtures, we move to consider the case of SI regimes. In Fig. 4 we plot the parameter Δ as a function of temperature for larger interactions, i.e. *U*/*t* = 20, *U*_*ab*_/*t* = 24, and *ω*_*H*_/*t* = 0.12. We find that Δ still decreases as the temperature increases. Due to the larger value of *U*_*ab*_/*t*, Δ drops by at least one order of magnitude less than in the WI case.

**Figure 5:**
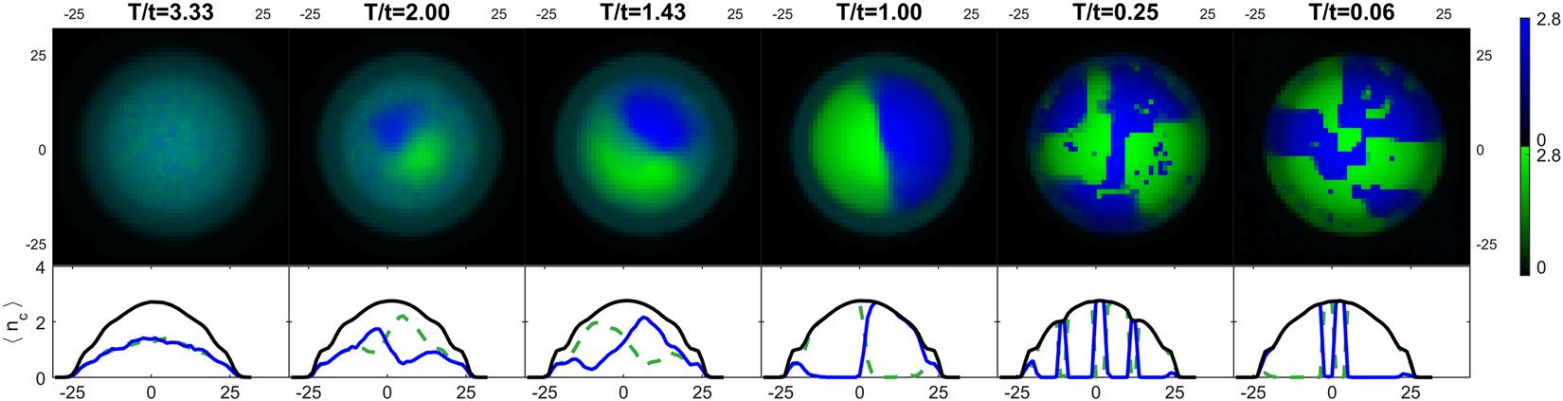
Density maps (first row) and density profiles (second row) of species A (green) and B (blue), for decreasing temperature (left to right) in the SI regime: *U*/*t* = 20, *U*_*ab*_/*t* = 24 and trapping potential strength *ω*_*H*_/*t* = 0.12. Density profiles 〈*n*_*c*_〉 are computed along the horizontal axis through the center of the trap. The total density profile is also shown (black-solid line).

Spatial shell structures arise also in the SI regime (see Fig. 5). For the values *U*/*t* = 20, *U*_*ab*_/*t* = 24 considered in Fig. 5, the phase diagram of the homogeneous case predicts different quantum phases depending on the filling. In particular, at zero temperature and integer filling, the system is expected to be a dMI. In the trapped case, such a phase can be observed in the regions of integer filling (density maps at *T*/*t* = 0.25 and *T*/*t* = 0.06 in Fig. 5). The strong interaction and very low temperatures are responsible for the fragmented structure of these density maps whose metastable character is discussed in the Supplementary information. Furthermore, at higher temperatures (left columns in Fig. 5) in the outer regions, we find a mixed phase forming a thin shell with a *n* = 1 plateau, suggesting the presence of a SCF phase in that region. The SCF at *U*_*ab*_ > *U* is made accessible by temperature excitations, since the energy separation between the dMI and the SCF is of the order of ~$|{t}^{2}/U-{t}^{2}/{U}_{ab}+U-{U}_{ab}|$ ^34, 35^.

## Experimental Realization

A mixture with *t*_*a*_ = *t*_*b*_ and *U*_*a*_ = *U*_*b*_ can be realized with ^41^K atoms in the two lowest hyperfine states $a=|F=1,m=1\rangle$ and $b=|F=1,m=0\rangle$ (it is understood that the hyperfine quantum numbers are used only as labels at high magnetic fields). In presence of a magnetic field ${B}_{0}\simeq 675\,{\rm{G}}$ the above mixture is predicted to feature a relatively narrow Feshbach resonance (*δB* = 0.15 G) between unlike states, while for like particles the scattering lengths are approximately constant across the narrow resonance and equal to each other (${a}_{a}\simeq {a}_{b}\simeq 60{a}_{0}$)^36^. Therefore, with a magnetic field near *B*_0_ it is possible to tune *U*_*ab*_, with minimal changes in *U*_*a*_ and *U*_*b*_. For a heteronuclear mixture of *a* = ^41^K and *b* = ^87^Rb in a square lattice, tunneling rates can be made nearly equal with an appropriate choice of the lattice step. For example, for a lattice step *d* = 380 nm, at lattice strengths such as 5 ≤ *U*_*b*_/*t*_*b*_ ≤ 30, we have 0.85 ≤ *t*_*b*_/*t*_*a*_ ≤ 1.15 and the ratio *U*_*b*_/*U*_*a*_ = 0.58 is constant.

The measurement of parameter Δ can be obtained directly from high resolution microscope images^8–13^. However, being a global observable, Δ does not require knowledge of the local densities and is also obtained by spectroscopic techniques. Indeed, the number of sites occupied by both species can be detected by driving transitions, sufficiently narrow in energy, towards excited states that can either be internal hyperfine states^37^ or external motional states, such as states of excited lattice bands^38^. In practice, the task is eased in proximity of the interspecies Feshbach resonance enhancing *U*_*ab*_. For sake of concreteness, we focus on microwave (or radiofrequency) transitions between internal hyperfine states, and we assume that for excited atoms the interatomic interactions are negligible with respect to *U*_*ab*_. Since *U*_*ab*_ is the energy cost required for the formation of a pair *AB* on the same site, then microwave photons will drive transitions in all lattice sites with occupation numbers $({n}_{a},{n}_{b})=(\ell ,m)$ or $(m,\ell )$ if their frequency is shifted by ~−${U}_{ab}\ell m$ with respect to the bare hyperfine splitting. Thus, the number of atoms in the excited hyperfine state as a function of the microwave frequency shows several different peaks, each corresponding to a specific pair of occupation numbers, $(\ell ,m)$ or $(m,\ell )$. The area of the observed peaks yields the relative number of the lattice sites, $f(\ell ,m)$, with the given fillings. Once $f(\ell ,m)$ values are known for all pairs $(\ell ,m)$, the demixing parameter is readily obtained as ${\rm{\Delta }}={\sum }_{(\ell ,m)}\,f(\ell ,m)(\ell -m{)}^{2}/(\ell +m{)}^{2}$.

Finally, we briefly discuss the accuracy of our proposed technique. For $u\equiv {U}_{ab}-U\gg t$, i.e. the regime where the above cited spectroscopy is to be performed, Δ decreases gently with *T*/*t*, enabling the use of the thermometer in a continuous fashion. In this range, the value of Δ is weakly dependent on *u*, thus the uncertainty on *u* negligibly affects that on *T*/*t*. In addition, since the measured value for Δ allows to infer *T*/*t* values, to convert the temperatures in SI units (i.e. kelvin), we are bound to introduce a relative uncertainty *δT*/*T* = *δt*/*t*.

The parameters of the Hamiltonian are known with satisfactory precision: interaction strengths can be determined with relative uncertainty below 10^−2^, see e.g. ref. 39, and similarly the tunneling rates^40^. Such levels of uncertainty are certainly tolerable for measurements of temperatures in deep optical lattices.

**Figure 6:**
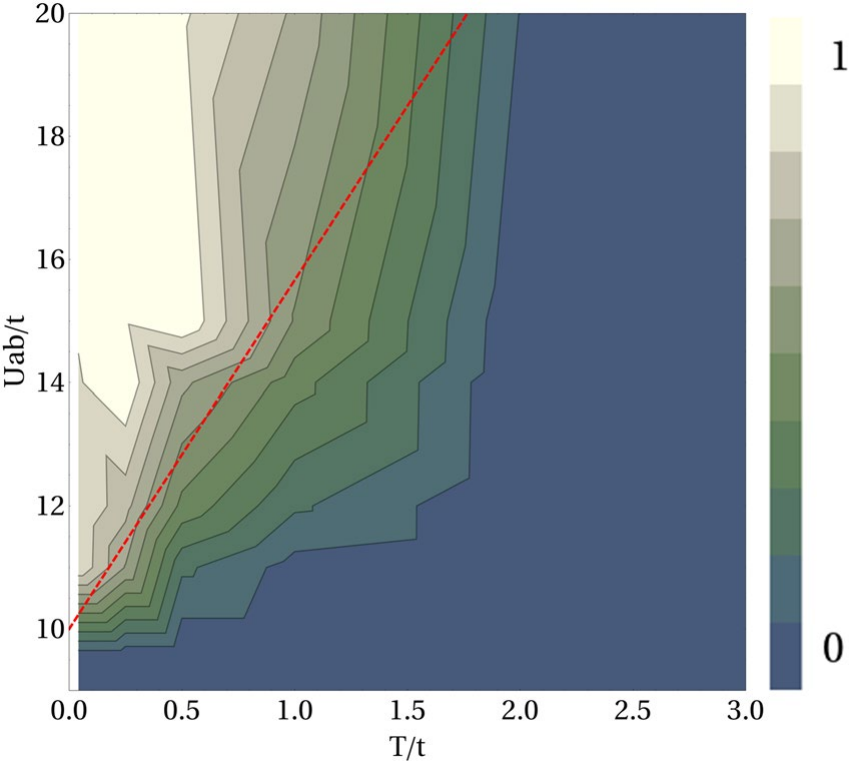
Contour-plot of Δ as a function of temperature *T*/*t* and interspecies interactions *U*_*ab*_/*t*, at *U*/*t* = 10. Total filling *n* = 2 and linear size of the lattice *L* = 24 (same data of Fig. 2, right panel). Red-dashed line shows critical boundary between the demixed and mixed phases computed theoretically through the relation ${k}_{B}T=\mathrm{2(}{U}_{ab}-U)/\mathrm{ln}({L}^{4}\mathrm{/4)}$.

For the sake of clarity, in Fig. 6, the right-panel of Fig. 2 is represented in a linear instead of logarithmic scale. This figure shows the contour-plot of Δ as a function of both *T*/*t* and *Uab*/*t*. One clearly sees how, for sufficiently large values of *U*_*ab*_/*t*, Δ exhibits a linear behaviour when *T*/*t* is varied in the interval [*T*_1_, *T*_2_] with ${T}_{1}\approx 0.5$ and ${T}_{2}\approx 2$. In Fig. 6 we also plot the critical boundary (red-dashed line) between the mixed and demixed phases predicted heuristically above (${k}_{B}T=\mathrm{2(}{U}_{ab}-U)/\,\mathrm{ln}({L}^{4}\mathrm{/4)}$). We notice that the simulated boundary is very well reproduced by the heuristical formula.

## Conclusions

We investigated temperature effects on the demixing of a binary mixture in both the homogenous and trapped case. Temperature fluctuations progressively destroy spatial separation between the two species with signatures visible also in the momentum distribution. Quite reasonably, higher fillings manifest stronger robustness against temperature-induced mixing. In both the weakly- and the strongly-interacting regime, the demixing parameter Δ is suppressed in a temperature range of the order of the tunneling energy. We therefore propose to use the experimentally measurable demixing parameter as a thermometer for strongly-correlated binary mixtures in the demixed phase. With the recent observations of anti-ferromagnetic correlations, reliable thermometry in optical lattices for strongly-interacting regimes is sorely needed to advance the field of ‘quantum magnetism’ with ultracold atoms.

## Methods

The investigation presented above has been carried out by performing simulations by means of the two-worm algorithm quantum Monte Carlo^32, 41, 42^, a Path-Integral technique that works within the grand canonical ensemble. It exploits the imaginary-time evolution to evaluate quantum-thermal expectation values of different physical quantities.

### The worm algorithm

According to quantum statistical mechanics the expectation value of a physical observable is given by

5 $$\langle \hat{O}\rangle =Tr(\hat{\rho }\hat{O})=\sum _{\alpha }\,\langle \alpha |\hat{\rho }\hat{O}|\alpha \rangle $$

where $\hat{O}$ is the quantum-operator corresponding to the physical observable *O*, $\hat{\rho }={e}^{-\beta \hat{H}}/{\mathcal{Z}}$ is the the density operator and ${\mathcal{Z}}=Tr({e}^{-\beta \hat{H}})$ the partition function. The parameter *β* = 1/*T* is the inverse temperature and is taken in unit of *k*_*B*_ = 1. The density operator can be treated as unitary evolution operator in imaginary-time *τ* = *i* · *t*. This allows to estimate the expectation value of a generic observable $\langle \hat{O}\rangle$ as an unitary evolution in imaginary-time between *τ* = 0 and *τ* = *β*.

Within the interaction picture, the imaginary-time evolution operator takes the form ref. 42:

6 $${e}^{-\beta \hat{H}}={e}^{-\beta {\hat{H}}_{0}}\cdot \hat{{\bf{T}}}{e}^{-{\int }_{0}^{\beta }\hat{V}(\tau )d\tau }$$

where $\hat{{\bf{T}}}$ is the time-ordering operator, and $\hat{V}(\tau )={e}^{{\hat{H}}_{0}\tau }\hat{V}{e}^{-{\hat{H}}_{0}\tau }$, while ${\hat{H}}_{0}$ and $\hat{V}$ are the diagonal and off-diagonal part of Hamiltonian $\hat{H}$, respectively. The time evolution operator $\hat{\sigma }=\hat{{\bf{T}}}{e}^{-{\int }_{0}^{\beta }\hat{V}(\tau )d\tau }$ in the interaction picture can be expressed in its iterative expansion form (the Matsubara time-evolution operator)

7 $$\hat{\sigma }=\hat{{\bf{T}}}{e}^{-{\int }_{0}^{\beta }\hat{V}d\tau }={\mathbb{1}}+{\hat{\sigma }}^{\mathrm{(1)}}+\cdots +{\hat{\sigma }}^{(n)}$$

where the n-*th* order term has the form

8 $${\hat{\sigma }}^{(n)}={(-\mathrm{1)}}^{n}{\int }_{0}^{\beta }\,d{\tau }_{n}{\int }_{0}^{{\tau }_{n}}\,d{\tau }_{n-1}\cdots {\int }_{0}^{{\tau }_{2}}\,d{\tau }_{1}\hat{V}({\tau }_{n})\hat{V}({\tau }_{n-1})\cdots \hat{V}(\tau \mathrm{)}.$$

The chain of operators $\hat{V}({\tau }_{n})\hat{V}({\tau }_{n-1})\cdots \hat{V}(\tau )$ describes the evolution of the system between *τ* = 0 and *τ* = *β*. By expanding the off-diagonal operator $\hat{V}$ in an operator basis

9 $$\hat{V}=\sum _{l}\,{\hat{K}}_{l}$$

such that ${\hat{K}}_{l}$ are hermitian and their action on a Fock state of the system results in another state of the same Fock space ${\mathcal H}$

10 $${\hat{K}}_{l}={\hat{K}}_{l}^{\dagger },\,{\hat{K}}_{l}|\alpha \rangle ={k}_{l\gamma }|\gamma \rangle ,\,with\,|\alpha \rangle ,|\gamma \rangle \in  {\mathcal H} .$$

The n-*th* order term of the imaginary-time evolution operator can be rewritten in the form

11 $${\hat{\sigma }}^{(n)}=\sum _{{l}_{n},{l}_{n-1},\ldots ,{l}_{1}}\,{(-\mathrm{1)}}^{n}{\int }_{0}^{\beta }\,d{\tau }_{n}{\int }_{0}^{{\tau }_{n}}\,d{\tau }_{n-1}\cdots {\int }_{0}^{{\tau }_{2}}\,d{\tau }_{1}{\hat{K}}_{{l}_{n}}({\tau }_{n}){\hat{K}}_{{l}_{n-1}}({\tau }_{n-1})\cdots {\hat{K}}_{{l}_{1}}({\tau }_{1}\mathrm{)}.$$

It is then possible to rewrite the trace (5) as

12 $$\begin{array}{rcl}\langle \hat{O}\rangle  & = & Tr(\hat{\rho }\hat{O})\\  & = & \frac{1}{{\mathcal{Z}}}\sum _{\alpha }\,\langle \alpha |{e}^{-\beta {\hat{H}}_{0}}\hat{\sigma }\hat{O}|\alpha \rangle \\  & = & \frac{1}{{\mathcal{Z}}}\sum _{\alpha }\,\langle \alpha |{e}^{-\beta {\hat{H}}_{0}}\hat{\sigma }|{{\rm{\Theta }}}_{\alpha }\rangle \\  & = & \frac{1}{{\mathcal{Z}}}\sum _{\alpha }\,\sum _{n}\,\sum _{{l}_{n},{l}_{n-1},\ldots ,{l}_{1}}\,{(-\mathrm{1)}}^{n}{\int }_{0}^{\beta }\,d{\tau }_{n}{\int }_{0}^{{\tau }_{n}}\,d{\tau }_{n-1}\cdots {\int }_{0}^{{\tau }_{2}}\,d{\tau }_{1}\\  &  & \times \langle \alpha |{e}^{-\beta {\hat{H}}_{0}}{\hat{K}}_{{l}_{n}}({\tau }_{n}){\hat{K}}_{{l}_{n-1}}({\tau }_{n-1})\cdots {\hat{K}}_{{l}_{1}}({\tau }_{1})|{{\rm{\Theta }}}_{\alpha }\rangle \end{array}$$

where $|{{\rm{\Theta }}}_{\alpha }\rangle =\hat{O}|\alpha \rangle$ is the Fock state resulting from the action of operator $\hat{O}$ on the state $|\alpha \rangle$. The expectation value of the observable *O* is then computed as a sum of all the possible evolution in imaginary-time from all the possible initial state $|{{\rm{\Theta }}}_{\alpha }\rangle$ at *τ* = 0 to the corresponding definite final state |*α*〉 at *τ* = *β*. The chain of operators ${\hat{K}}_{{l}_{n}}({\tau }_{n}){\hat{K}}_{{l}_{n-1}}({\tau }_{n-1})\cdots {\hat{K}}_{{l}_{1}}({\tau }_{1})$ defines the path in imaginary-time (i.e. worldline) from state $|{{\rm{\Theta }}}_{\alpha }\rangle$ to the state |*α*〉. Since the whole computation of ${\mathcal{Z}}$ and the generation of all the possible paths would be computationally too costly, a Monte Carlo sampling is used to sample only those paths that contribute the most to the expectation value. The Monte Carlo algorithm generates at each Monte Carlo step a different configuration, and, via a Metropolis Method, accepts or rejects it with a probability that satisfies a proper detailed-balance equation^41, 42^.

The states of the system described by the two-species Hamiltonian (1) is the tensor product of the Fock states in the spatial mode representation of the two bosonic species

13 $$|\alpha \rangle =|{\alpha }_{a}\rangle \otimes |{\alpha }_{b}\rangle =|{n}_{a1}{n}_{a2}\ldots {n}_{aM}\rangle \otimes |{n}_{b1}{n}_{b2}\ldots {n}_{bM}\rangle $$

where |*α*_*a*_〉 and |*α*_*b*_〉 are the Fock states of species A and B respectively, and *n*_*ci*_ the *i*-site occupation number of species *C* = *A*, *B*.

The worm algorithm^41, 42^ works in an enlarged configuration space by introducing a disconnected worldline, the worm. This results in working in the grand-canonical ensemble, where particles can be added/removed to/from the system. “Head” and “tail” of the worm destroy and create a particle in a given site *i* and imaginary time *τ*. They correspond to the annihilation operation *c*_*i*(*τ*)_ and the creation operator ${c}_{j(\tau )}^{\dagger }$ respectively. Consequently, when the worm is present in the cofiguration eq. 12 refers to the Green function: $G(i,j,\tau )=\langle {\hat{{\bf{T}}}}_{\tau }{c}_{i}(t+\tau ){c}_{j}^{\dagger }(t)\rangle$.

Through operators ${\hat{K}}_{l}({\tau }_{m})$, head (*c*_*i*(*τ*)_), tail (${c}_{j(\tau )}^{\dagger }$) the worm moves in space and imaginary time (jump, reconnection, shift in time …^41, 42^) thus generating new configurations. In our case, in order to explore the configuration space of both the two bosonic species, we use two independent worms that act respectively on the Fock space of the two bosonic species^32^.

### Estimation of Quantum-Correlators

To compute the momentum distributions and to check the superfluid/normal-fluid phase transition we estimated quantum-correlators of the form $\langle {c}_{i}^{\dagger }{c}_{j}\rangle$. The computation of quantum-correlators $\langle {c}_{i}^{\dagger }{c}_{j}\rangle$ through the 2-Worm-Algorithm is achieved by collecting statistics of the position of “head” and “tail” of the worm. Every time the “tail” (${c}_{i}^{\dagger }$) and the “head” (*c*_*j*_) of the worm of species *C* = *A*, *B* are found in position *i* and *j* respectively, the correlation-matrix element *M*_*cij*_ is increased (*c* = *a*, *b*). The quantum-correlators are then estimated as

14 $$\langle {c}_{i}^{\dagger }{c}_{j}\rangle \propto {M}_{cij}.$$

### Simulation Setup

Each simulation is carried out by setting both the temperature and the number of particle. The worm-algorithm quantum Monte Carlo works within the picture of the grand canonical ensemble at finite temperature. The temperature is controlled through the inverse-temperature parameter *β*. The number of particles in the system is controlled via the chemical potentials *μ*_*a*_ = *μ*_*b*_ = *μ*. By carefully tuning the value of *μ* it is possible to control the total number of bosons in the lattice. However, controlling the number of particles of each species turns out to be challenging. This leads to population densities which varies during the Monte Carlo time. For example, what typically happens in the regime of the demixing effect is a depletion of one of the bosonic species as the Monte Carlo time goes by ref. 27. This situation has to be avoided as in real experimental setup one is capable of controlling independently the number of bosons of each species with a finite precision. In order to avoid this problem and ensure the conservation of particle of each bosonic species during the simulation we restricted the Hilbert space of the grand canonical ensemble by introducing an upper bound in the quantum fluctuation of the number of particle of each species. This procedure allows us to keep balanced the populations of the two species ${N}_{a}\approx {N}_{b}\approx N/2$.

### Data Availability

The datasets generated during the current study are available from the corresponding author on reasonable request.

## Electronic supplementary material

Supplementary information: Thermometry of bosonic mixtures in Optical Lattices via Demixing
