# Supplementary material for: Thermometry of bosonic mixtures in Optical Lattices via Demixing
Source: Sci Rep. 2017 Jul 11;7:5105. doi: 10.1038/s41598-017-05353-6 (PMC5506072; doi:10.1038/s41598-017-05353-6)
Supplement: Supplementary file 1 — Supplementary information: Thermometry of bosonic mixtures in Optical Lattices via Demixing [file 41598_2017_5353_MOESM1_ESM.pdf]

Supplementary information: Thermometry of bosonic mixtures in Optical Lattices via Demixing

F. Lingua[1,*], B. Capogrosso-Sansone[2], F. Minardi[3,4], and V. Penna[1]

[1]Department of Applied Science and Technology and u.d.r. CNISM, Politecnico di Torino, I-10129 Torino, Italy
[2]Department of Physics, Clark University, Worcester, Massachusetts 01610
[3]Istituto Nazionale di Ottica (INO-CNR)
[4]European Laboratory for Non-Linear Spectroscopy (LENS) and Dipartimento di Fisica, Università di Firenze, I-50019 Sesto Fiorentino - Firenze, Italy
*fabio.lingua@polito.it

ABSTRACT

We report here supplementary information to our study on "Thermometry of bosonic mixtures in Optical Lattices via Demixing". The following discussion mainly concerns the harmonically trapped case in which we observe the appearance of the spatial shell structures.

Spatial-shell structure and momentum distributions

As shown in the main paper, increasing temperatures leads to a progressive destruction of the spatial demixing between the two species. This effect first appears in those regions where the entropy associated with demixing is larger (outer shell where the density is lower and in proximity to the boundary between the two species). However, it is not restricted only to those region but it extends to the entire lattice. This is shown in the lower row of Fig. 1: in the case of shell structure (left panel) where the density of one species reaches its maximum, the other does not go identically to zero, as in the completely demixed case (right panel). A partial but finite mixing of the two species is present everywhere in the lattice.

As far as the momentum distribution is concerned, by increasing the temperature in the system we observe an abrupt change marking the appearance of the spatial-shell structure and the mixed phase. This dramatic change is outlined in Fig. 2, where we plot together density and momentum distribution of both species for a spatial-shell structure configuration at $T/t = 1.00$ (left column) and a completely demixed one at $T/t = 0.06$ (right column). At low temperature, we observe oscillations/fringes in the momentum distribution, essentially due to the interference of waves bouncing from the "hard-wall" separating the demixed species. The oscillations/fringes disappear as soon as the mixing deconfines the two species and phase coherence is restored among all the lattice sites.

Momentum distributions of metastable states

By using the momentum distributions it is possible to understand if the demixed system is in the ground state configuration or in a metastable state close to it. In the WI regime, the minimum-energy configuration in a harmonically-trapped systems features a straight boundary between the two species[1] (see Fig. 2, right panel). We note, however, that in demixed phases of Bose-Hubbard models[2,3] there exists infinitely-many local minima featuring metastable states very close in energy to the ground state. The corresponding configurations of the mean occupation numbers slightly differ from the ground state and feature, in general, irregular or multiple boundaries between the two species. Numerical simulations suggest that this kind of configurations are also observed in the SI regime where, for integer local filling, fragmented dMI regions can be stabilized (see Fig. 5, main text).

By computing the momentum distributions of such metastable configurations, we find that the irregular/multiple boundaries exhibited by the demixed states lead to irregular and more complex interference patterns. Two example of these configurations are shown in Fig. 3.

Normal fluid Phase Transition

For sufficiently high temperatures, the outer (spatial) shell is filled with a normal fluid (NF). In order to check the presence of the NF phase we computed the field-field correlators:

$$\langle \Psi_c(r)^\dagger \Psi_c(d) \rangle = \sum_{i:|\mathbf{r}_i|=r} \sum_{j:|\mathbf{r}_i-\mathbf{r}_j|=d} \langle c_i^\dagger c_j \rangle \tag{1}$$

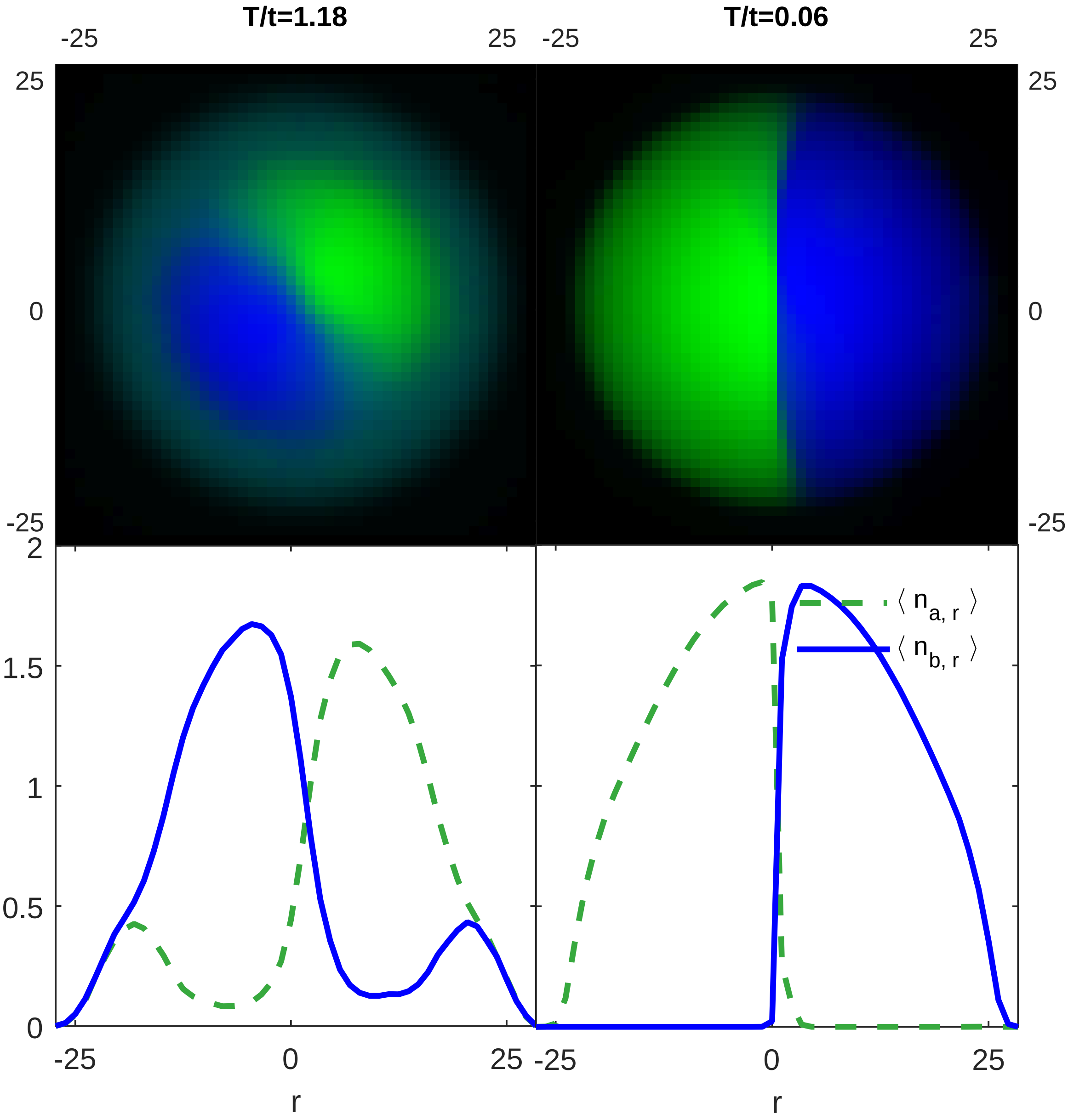

Figure 1. Density maps (first row) and their section at $y = L/2$ along x-direction (second row) for temperatures $T/t = 1.00$ (left column) and $T/t = 0.06$ (right column). The mixture ($U/t = 10$, $U_{ab}/t = 15$) is trapped in a harmonic potential of strength $\omega_H/t = 0.03$.

for species $c = a, b$. These are obtained from correlators $\langle c_i^\dagger c_j \rangle$ by grouping together all the sites i at a given radius r (from the center of the trap) and all the sites j at a given distance $d = |\mathbf{r}_i - \mathbf{r}_j|$ from the sites i. In the right column of Fig. 4 we show the behaviour of correlators (1) of species A for increasing temperatures. Due to symmetry, the correlators of species B manifest the same behaviour and therefore are omitted. For the sake of completeness, the left column of Fig. 4 displays the corresponding density maps. Fig. 4 shows that for low temperatures the field-field correlation is spread uniformly all over the occupied lattice sites. On the other hand, we notice that for sufficiently high temperatures the system manifests long-range correlation in a disk-like region around the trap center, and short-range correlation for larger radial distances. In particular, long-range correlations suggest the presence of superfluid phases (dSF and 2SF) in the central disk-like region, while short-range correlations suggest the presence of an external NF shell in the outer portion of the lattice. We notice as well that

spatial-shell structures features an increasing thickness of the NF shell for increasing temperatures (last two rows of Fig. 4).

References

1. P. Ao and S. T. Chui, Phys. Rev. A 58, 6 4836 (1998)

2. P. Buonsante, S. M. Giampaolo, F. Illuminati, V. Penna, and A. Vezzani, Phys. Rev. Lett. 100, 240402 (2008)

3. T. Roscilde, and J. I. Cirac, Phys. Rev. Lett. 98, 190402 (2007)

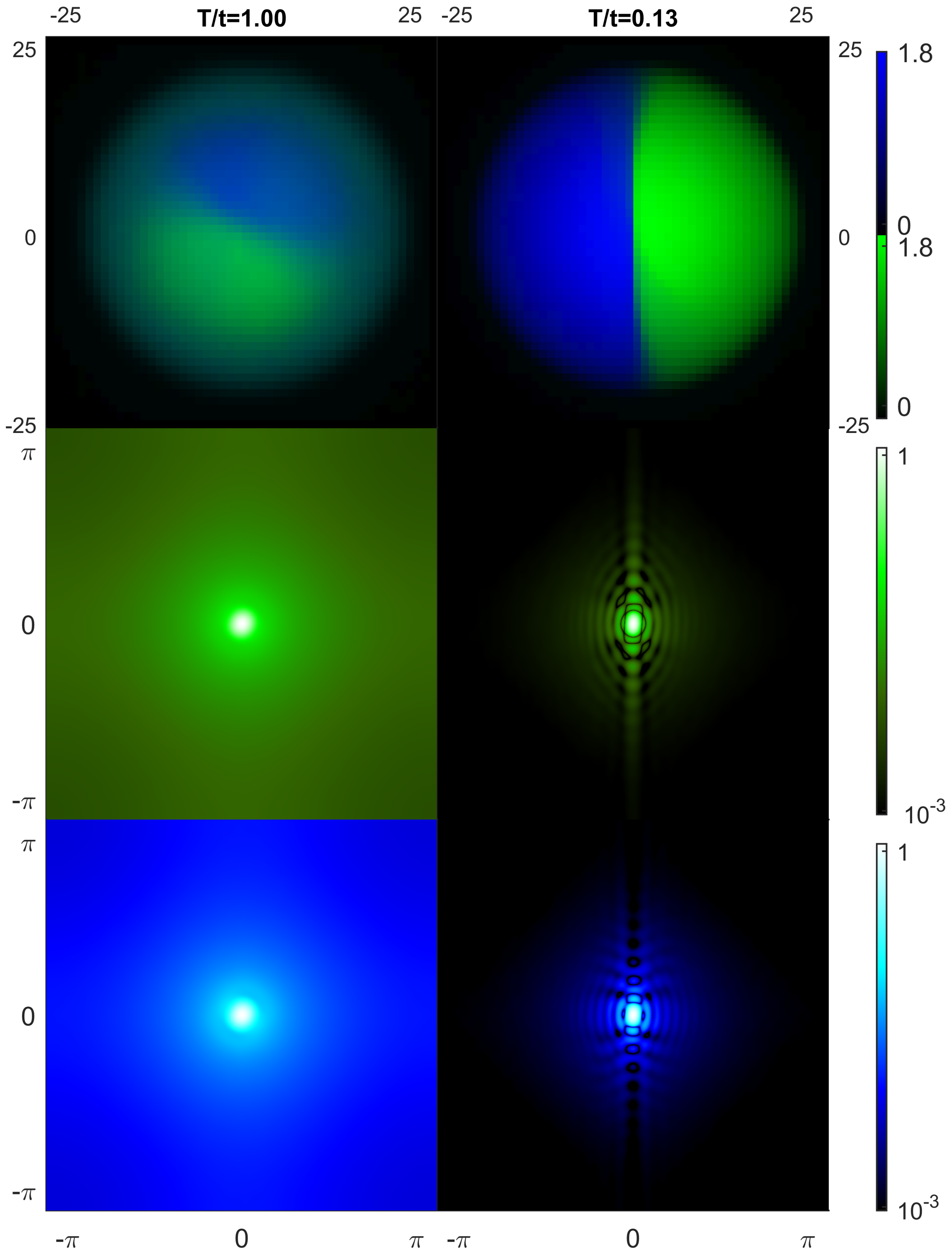

Figure 2. Density maps (first row) and computed momentum distributions for specie A (second-row) and B (third-row) for temperatures $T/t = 1.00$ (left column) and $T/t = 0.06$ (right column). The mixture ($U/t = 10$, $U_{ab}/t = 15$) is trapped in a harmonic potential of strength $\omega_H/t = 0.03$.

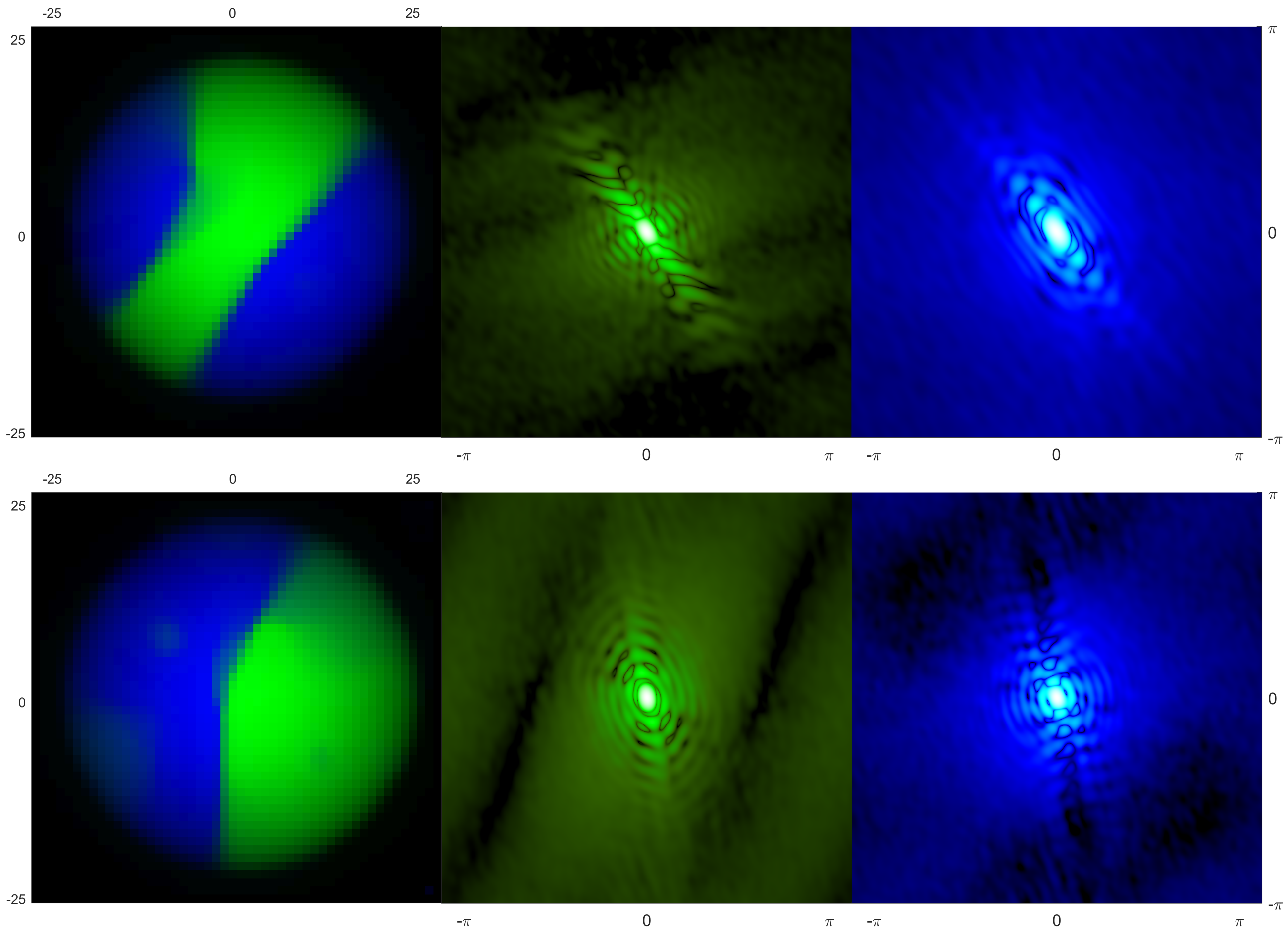

Figure 3. Metastable configurations and related momentum distributoins of species A (central column) and B (right column). Simulation performed with $N \approx 1453$ (Upper panel) and $N \approx 1644$ (Lower Panel) at $T/t = 0.042$, $U/t = 10$, $U_{ab}/t = 15$ and $\omega_H/t = 0.03$

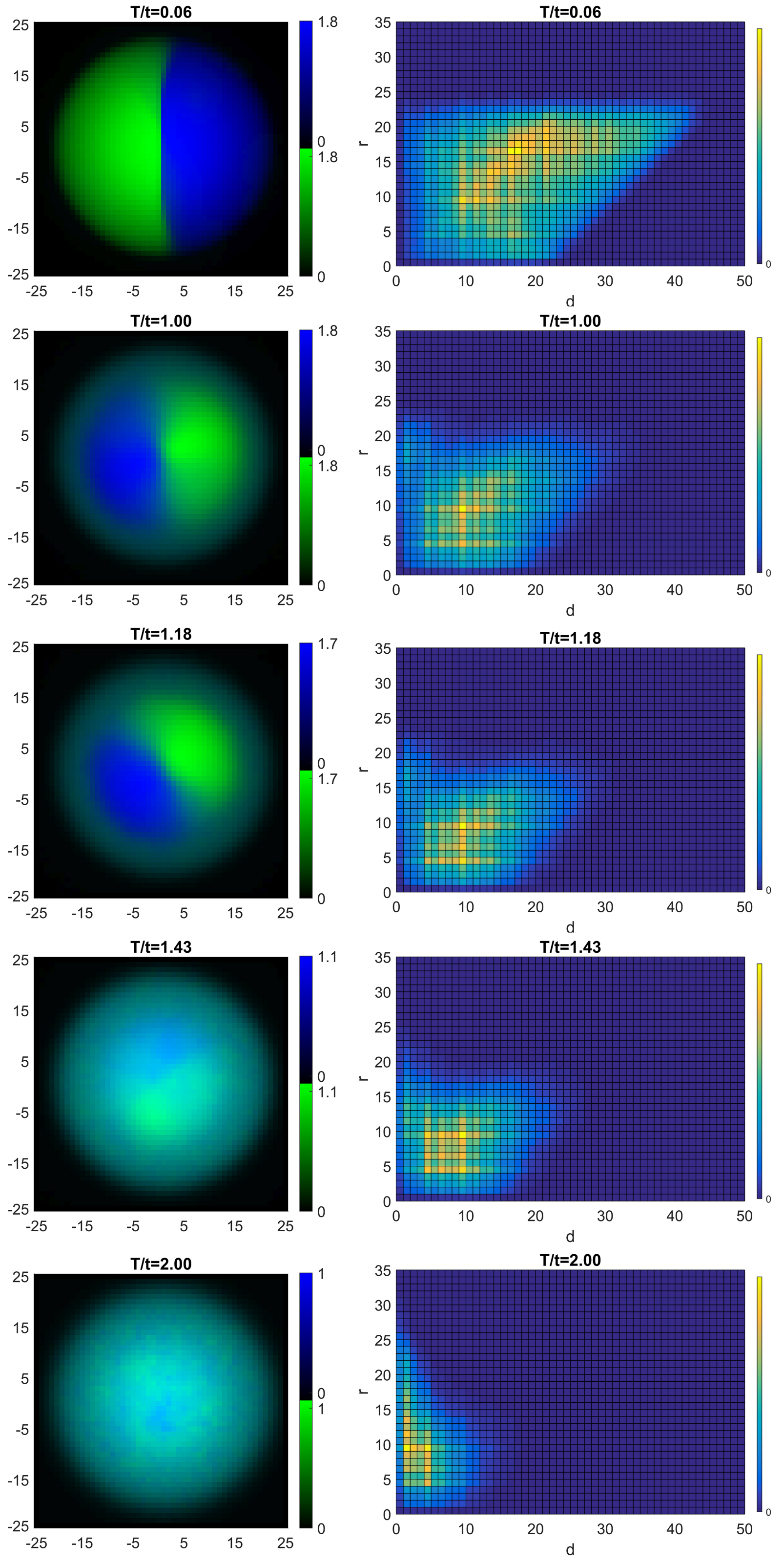

Figure 4. Field-Field correlators (in arbitrary units) of a single bosonic species for increasing temperatures (right column), and their associated density maps (left column).
